# The Nutritive Functions

**Published:** 1884-12

**Authors:** A. T. Douds

**Affiliations:** Canton, O.


### The Nutritive Functions.

BY DR. A. T. DOUDS, CANTON, O.

That mysterious union of an unknown force with matter which we term life, Bichat defines as "the aggregate of the functions which resist death," and which he classified under two heads, viz.: organic and mineral life. The first comprehends the various functions concerned in maintaining this union, whether during assimilative or retrogressive change; the second, those functions which distinguish man and animal from other forms of life.

The maxim of Harvey, "*Omnia viventu ex ovo*," all life springs from an egg or cell, stands uncontradicted, and in which cell, the combination of heat, light, and force obtained from without, reappears in the form of vital force, which, like all other forms of force, only shows the dynamic conditions through which the integrity of the parts is maintained.

In the cell we find "the ultimate physical elements of organization," and being endowed with life, and an independent and definite period of existence, unless destroyed or hardened, it dies at the expiration of its time and is cast off.

Each molecule entering into the composition of organized existence, though composed of elements as old as creation, by being brought into this new relation has the power, under the guidance of its "*vis essentialis*," of so acting upon certain kinds of matter as to reproduce itself indefinitely, as well as the faculty of effecting deffinite cycles of change in form and composition, in which the type as well as certain characteristics are reproduced.

Professor Goodsir says: "The entire organism is formed first, not by the simultaneous formation of its parts, but by successive development of these from one center, this being the original source of all centers with which the part is ultimately supplied," and this series of successive phases through which every part of the living body passes, indicates an inherent capacity for growth, which gives rise, primarily, to the demand for nutrition, which is but a continuous molecular process, whether in the zoophyte, one of the lowest forms of organized existence, or in the more complex structure of man.

Eminent microscopists describe the cell as composed of a nucleus —which is the "germinal matter" of Beale, the "sarcode" of Dujardin, or the "protoplasm" of Max Schultze—which is centrally located, and upon which its origin, as well as its existence depends; and the outer substance which Beale describes as "having passed through the germinal and vitalizing stage, takes its place as 'formed matter' outside of the nucleus."

To quote from the latter, in describing growth of tissue, he says: "Pabulum becomes germinal matter, while germinal matter is converted into new formed material, which is added to that which already existed, and unless condensation, drying, or shrinking occurs to compensate for the addition, increase of bulk and growth of tissue must take place."

The organism is composed of a variety of tissues, presenting great diversity of character, and it is the definite function of each molecule to select, assimilate, and build up out of the material held in solution by the serum of the blood, which also forms the medium of its conveyance to the tissues, those constituents which furnish the special pabulum for the development and reparation of the several parts, and by that inscrutable "chemico-vital" process, transforming it into living tissue.

In the development of animal life two forms of substances are required, viz.: organic and inorganic, both being found in due amount in all ordinary forms of diet, the relative value of different substances as food being measured by their tissue-forming and heat-producing qualities, the amount of albumen which they contain determining their histogenetic value, and the hydrocarbonaceous matter determining their value as heat-producers.

Albumen is found in abundance in both the animal and vegetable kingdoms, and aside from its histogenetic value possesses remarkable properties which render it indispensable to the animal economy. Readily soluble in water, it possesses not only the property of preserving the fluidity of the blood, and facilitating its passage through the capillaries, but also that of so combining with the nutritive material taken into the circulation as to blend its various elements into a homogenous mass upon which the "elective affinities" of the various tissues may exert their force, selecting and appropriating that which they may require without interfering with the remaining constituents.

The degree to which albumen is capable of so affecting the chemical relations of substances in the blood as to prevent the formation of new compounds which otherwise might result from digestive decomposition, except that which occurs through "chemico-vital" action in the tissues, is remarkable.

Carpenter states that "such is its influence that lactate of iron mixed with the blood is so thoroughly disguised that cyanide of potassium fails to produce the Prussian blue until after the albumen has been destroyed by the action of an acid. This property appears all the more striking when, according to a proximate analysis of blood, given by Simon, its complicated nature is shown by its including forty-two different constituents, three only being elemental and the remainder each representing a compound.

According to the best authorities, this substance is found to be a constituent of all serous fluids, and Paget says, "mucous secretions consist of epithelium cells and mucous corpuscles, the latter of which are greatly increased by *irritation* of the mucous membrane accompanied by an *excess* of albumen;" and according to

Carpenter, when found in the secretions is abnormal and is an evidence of some derangement.

As to the influence of the decomposition of this substance, in the occurrence of decay of the teeth, when found in connection with the saliva, mucous secretions, and serous exudations from diseased gums, slight reference will be had farther on.

In the numerous analyses of bone, tooth, ligament, brain, spinal cord, muscle, etc., by many investigators, so uniformly is the presence of certain inorganic substances found as to compel their recognition as essentially histogenetic in their character, the phosphate and carbonate of lime being ever present, in varying proportion, according to the character of the tissue. The carbonate, according to M. Edwards, existing, however, for the most part, "only as a product of the decomposition of the phosphate, brought on by the fluids of the organism during chemical combination of the *basic* phosphate of lime and gelatine which forms bone."

Lehman regards the phosphate of lime as by far the most important of all the mineral substances "which are of service to the animal body by their 'physical properties.'"

Simon, in his estimate of its importance to the animal organism, terms it next to water, and gives the proportions of lime and phosphoric acid as 52 to 48.

Chosat, by restricting animals to food containing little or no phosphate of lime, succeeded in producing artificial softening of the bones, affording not only evidence of its histogenetic importance, but establishing the fact that, in the process of cell disintegration, the lime salts are removed, and when not renewed present the above results, bone nutrition being completely interstitial, disintegration, like assimilation, being effected throughout its substance.

According to Simon, in most pathological conditions the quantity of lime salts excreted, decreases, yet, in an analysis of the urine of a child, given by Berzelius, who was being treated for rachitis, of 38 parts of solid residue out of 1,000, 18 parts were fixed salts and earthy phosphates, and he adds, "on calculating the ratio of these constituents to 100 parts of solid residue, and comparing them with those that occur in the healthy secretions, we find the quantity of urea has decreased, while that of the salts has increased, especially the phosphate of lime," showing not only a failure of the tissues to assimilate what was ingested in the food, but also an interstitial waste in excess of that occurring in health.

An analysis of healthy urine, by Simon, showed 16 parts of fixed salts, and $1\frac{1}{2}$ of earthy phosphates to 100 of solid residue; and by Lehman and others of various ages, $1\frac{1}{2}$ of phosphate of lime and magnesia, and about the same proportion of other constituents as given by the former.

In a report the Academy of Science, Doune stated that the urine, during pregnancy, contained a decreased amount of phosphate and sulphate of lime, which was not confirmed by Becquerel, Simon, and Witthaus, who also give a number of analyses, in which but little variation occurs from that of ordinary healthy urine, while it is a well-known fact that fractures occurring during pregnancy, unite slowly, and occasionally not at all, which would indicate a deficiency of this substance.

From these observations it may be fairly concluded that, under all ordinary conditions of health, there is a continual waste of this substance from the tissues, the result of molecular death and disintegration, and this being true under the condition referred to, as well as during that of growth and development, when the demand for the phosphate of lime is especially great, and notwithstanding the statement of writers that the lime product of broken down tissue, even that of the roots of the deciduous teeth, is reappropriated. By the elimination of this salt during the time of its greatest requirement in the system, it would seem to have lost some essential principle during its residence in the tissues which renders it unfit for reappropriation.

The saline compounds in the urine, according to Carpenter, "are to be regarded as proceeding from the retrograde metamorphosis of the materials of the tissues after they have served their purpose in the economy, and partly from the components of food which was not organized, the small amount of carbonate of lime taken into the system with food being eliminated almost entirely in the form of the phosphate."

The saturating capacity of phosphoric acid renders it peculiarly adaptable to the metamorphosis of tissue, as upon the authority of Lehman, it can combine with one, two, or three atoms of base, the latter being a neutral phosphate.

Wilthaus also describes the phosphate of lime as existing under three combinations, the monocalcic, the dicalcic, and tricalcic phosphates, the first two being acid, and the tricalcic being neutral or bone phosphate, and having a remarkable "yielding property" to the weakest acids, even in its organized state, well accounts for the readiness with which the lime salts are dissolved out of the osseous tissues interstitially, or by the action of external agents.

Lehman sustains the conclusions of numerous observers, in the statement that " the phosphate of lime is formed within the animal organism, from its proximal constituents in the tissues, the alkaline phosphates introduced by grain, flour, beans, peas, etc., being decomposed by the action of sulphuric, lactic, hippuric, uric, and other acids which result from decomposition within the animal body, so that the liberated phosphoric acid must combine with the lime taken into the body with vegetable food, forming the phosphate of lime found in the various tissues."

The process of the formation of this salt within the animal tissues can be almost directly observed in the development of the chick in the egg; and it was Prout who first called attention to the constancy with which the phosphoric acid is maintained, while that of the lime is greatly augmented, the carbonate being transferred from the shell, its most proximate source, the phosphoric acid combining during inoculation—the shell decreasing greatly in weight—to form the phosphate of lime found in the bones, claws, feathers, etc.

The therapeutic influence of the various phosphatic compounds in certain pathological conditions, and the estimates placed upon their value by eminent physicians, is quite variable.

Dr. J. F. Churchill, in 1848, suggested the theory that the immediate cause of tuberculosis was a lack of the phosphates in the system, and he prescribed the hypophosphites, as being more soluble, in numerous cases with advantage.

Beneke regards the phosphate of lime as useful in incipient tuberculosis, and a number of other diseases, but found it of no service in caries of the bones in adults.

Dr. Colton, in the London hospital for consumptives, found it of no avail; and, after a three-years' test, concluded that it exerts no specification, and even when useful, found it inferior to other remedies.

M. DeChambre, of Paris, renders a similar verdict.

Mr. Taylor, of Liverpool, claimed for its action in work-house patients, especially in exhaustion from prolonged lactation, dentition in strumous children, and some other diseases, great advantage.

Whatever may be its specific value in certain pathological conditions, the influence exerted by the general stimulant effect accorded it by Stille and others, especially upon the nervous, muscular, and circulatory systems, is of undoubted value, its almost universal presence in the tissues facilitating, by the law of chemical affinity, its therapeutic influence.

The dentinal tubuli, opening into the pulp chamber, and radiating throughout the entire substance of the dentine, furnish a most admirable system of conduit for the conveyance of nutritive matter, and that such movement of nutrient fluid actually occurs, reference is made to the well-known gradual tinging of the teeth of animals from feeding madder.

The inter-tubular tissue, when highly magnified, is seen to be made up of minute granules closely united together through the medium of the organic constituents of the dentine; and it was Kolliker, if we are not mistaken, who first called attention to what he termed "inter-globular spaces," which he regarded as sustaining the same relation to the dentine that the lacunæ do to the nutrition of bone. The tubuli he describes as opening into these spaces.

Tomes describes them as "open, irregular spaces, frequently long and wide, and crossing a number of the tubuli, being most frequently found in teeth lost early in life, notably the first permanent molars, and filled with a cartilaginous substance analogous to the basis substance in which dentine is formed," but owing to some defect in the assimilative process, has failed to undergo calcification.

Prof. McQuillen, in 1866, was the first to call attention to the fact that the "inter-globular spaces" were not cavities, like bone lacunæ, being entirely dissimilar, and not distributed with the regularity which characterize the latter, being found in great numbers throughout the dentine, and proving that no such function is assigned them as was supposed by Owen and Tomes, and that instead of the tubuli terminating in these spaces, they continue their course directly through them.

The "contour lines" described by Owen, passing over the coronal portion of the tooth, as well as found in the root, are, according to Prof. McQuillen, identical with the "inter-globular spaces," subsequent calcification or filling up having taken place, the appearance under the microscope, leaving no doubt as to the secondary character of the deposit.

In the molecular changes occurring in the osseous system through the "physical properties" of the lime phosphates and carbonates, is furnished a clear illustration of the process of assimilation and disassimilation, as indicated in the formation of the frontal, and sphenoidal sinuses in children, absorption of the roots of the deciduous teeth, calcification of the "inter-globular spaces," filling up of the pulp chambers, increased density of the teeth, and thinning of the cranial bones in old age.

"The more active the normal nutrition of any tissue the more speedily does its molecular death follow the withdrawal of the conditions upon which its vital actions depend."—CARPENTER.

In the application of the converse of this statement to the dental tissues, certain physical phenomena are manifested synonymously with any interference with their normal nutrition, which, acting externally and in concert with the interstitial disintegration, results in the rapid breaking down of the teeth, the external influence being due, *indirectly*, to the vitiated condition of the oral secretions--farther facilitated by the structural defects or peculiarities referred to--and induced by nervous depression, the result of disease or exhaustion of vital force.

Bearing in mind the reference made to the importance, histogenetically, of a due supply of the inorganic constituents, more especially the phosphate of lime, the circumstances of a constitutional character, under which these conditions are commonly met and in which the demand for this substance is peculiarly great, are during the periods of gestation, also that of puberty, or at the age of from 12 to 18 in both sexes, in which rapid growth, and development, together with the constitutional changes peculiar to this time, create a demand upon the vital powers, which, if not met, must result more or less disastrously to these importtant organs, the teeth.

The pre-existing structural defects, the result of malassimilation, as exhibited in the formation of the inter-globular spaces, as well as the more externally manifested regions or transversely grooved and pitted condition, which Bourdet, as long ago as 1757, attributed to defective nutrition, the result of disease peculiar to childhood as: measles, scarlatina, rachitis, scorbutis, variola, etc., all predispose to decay from the action of even slight external agents.

If it be conceded that one of the conditions of the normal nutrition, the maintenance of the form, size, and composition of all organic bodies, is a due supply of food containing the elements upon which the vital forces may expend their energies, in the preservation of the harmony and integrity of the whole, of no less importance is the fact, that the animal economy will appropriate no greater amount than the vital principle, which stimulates the organ, will allow.

In the application of this truth it may be stated, also, that the relation existing between the nutritive functions and the various organs, bears a direct relation to the nervous organization of the individual, and that in the scale of excellence, nothing higher can be evolved, than is represented by the degree of nervous force in the animal system in which they are organized.

Hence, it may readily be seen that the diet may contain all the elements for the most perfect nutrition of every part, and yet interstitial decay may not be compensated: for by lack of power to assimilate, some persons thrive upon a barely sufficient and frugal diet, while others are reduced and become emaciated, although taking an abundance of nutritious food.

Living upon the same diet and under the same hygienic influences, members of the same family, present, in the structural character of the teeth, differences, which can be accounted for upon no other hypothesis; and that special organs should exhibit this tendency to decay, furnishes a pathological enigma difficult of solution; and in it we are compelled to recognize the impress of a pre-existing, pre-determining cause in which there is not merely an admixture of the "normal characteristics" in the offspring, but also the hereditary transmission of "perverted functional" activity which may have been peculiar to either or both the parents.

In the millions of cells which compose the tissues of the body, is located nature's great laboratory, in which, by her own immutable laws she adopts her own plans, her own time, and her own means of vitality, and no medication, no artificial nutrition, can control or change them.

Temporary advantage only may be gained by forcing her; and of this we may safely avail ourselves, when in harmony with her laws we intelligently supply the means through and upon which she may exert her forces, ever remembering that all her energies in "the aggregate of the functions which resist death," are silently, yet unceasingly, being exerted in the effort to maintain the harmony and integrity of every part.

And, as in the cell exist the "ultimate elements of organization," any functional disturbance must decrease the demand for the histogenetic elements; and whatever is in excess of this demand, either ingested with food, or administered therapeutically, can only result in harm, nature effecting its removal by purgative action or otherwise.

Liebig says, "A certain amount of cells is absolutely necessary to constitute normal blood; and the physical condition of the tissues oppose an obstacle to any increase or decrease of the amount of salts, and thus it cannot become richer or poorer in salts beyond a certain limit; and fluids containing a larger amount of salts than the blood, remain unabsorbed and leave the organism, through the rectum; and fluids containing a smaller amount than the blood enter the circulation, and are eliminated through the urinary channel, so that finally only those substances remain which exist in chemical combination with the constituents of the blood." *The richness of the blood in these elements is controlled by the active demand existing in the tissues.*

If the foregoing observations be based upon correct physiological principles, the utter futility, not to say absurdity, of any attempt to supply the indicated want by medication, is apparent, when it is safe to say that, except in exceedingly rare instances, there is a sufficient quantity of the lime salts in ordinary quantity and quality of daily food to subserve all the requirements of the nutrition of the body, but which, for want of vital force, the result of disease or some functional peculiarity, hereditary or acquired, there is a failure to assimilate.

In a large percentage of the cases coming under the notice of the dentist, the attempt to preserve those important organs, the teeth, by purely local and mechanical means, when the conditions upon which their very existence depends, remain imperfect, cannot but result more or less disastrously, either in their utter loss, or marred appearance and usefulness. The great benefit claimed from the use of the phosphates is, without doubt, due mainly to their general stimulant effect upon the nervous, muscular, and circulatory systems, to which reference has been made; and doubtless in the phosphatic preparations we possess an agent of great therapeutic value; yet its indiscriminate use, even in cases where it seems to be indicated, especially when exhited for the specific purpose of supplying the tissues with the phosphate of lime, is not only questionable practice, but not unattended with danger. In a work on medical chemistry, by R. M. Witthaus, published in 1881, he says, in speaking of the uses of this substance: "It is not improbable that recently introduced medicinal preparations containing phosphorus, may produce fatal poisoning in the future."

A consideration of no less importance, in the treatment of these cases, is the influence of a due amount of exercise, as well as fresh air: for, according to the observations of Berzelius, subsequently confirmed by Liebig, the presence of free lactic acid in the tissues is in proportion to the *extent to which they have been exercised;* and so great is the quantity, that the latter expressed the opinion that "it is more than sufficient to saturate all the alkaline fluids of the body."

The hygienic influence of exercise in developing this acid, considered in connection with the almost universal presence of the phosphate of lime in the tissues, and its solvent effect upon this substance, by obtaining for it a larger solution by decomposing it and liberating the phosphoric, whereby new combinations are possible, thus bring into direct relation with the tissues this important element of their nutrition, with their specifically stimulant effect, to which reference has been made, can scarcely be overestimated.

And in order that the various substances concerned in the nutrition of the body, requiring oxidation, be thoroughly effected —phosphorus being neither appropriated, nor eliminated until after having undergone thorough oxidation—the importance of an abundance of pure air, more constant and free than the sunshine, should be impressed upon the minds of our patients, and as far as our influence may extend, the thorough ventilation of school-rooms and dwellings, should be insisted upon; and so thoroughly should we be imbued with the true spirit of professional progress, so alive to the interests of our patients, freely diffusing the light of which we may be possessed, that, like bread cast upon the waters, it may return to us in that recognition of our counsel, which is accorded to the medical profession.

And believing that we are entering upon the dawn of the day, in which under clearer light, the local and mechanical, in the science of conservative dentistry, shall occupy their true positions only as valuable and indispensable adjuncts, and when, as an educated profession, the intelligence and skill displayed in our specialty, should *command* the respect of the world, then, and not until then, may we hope for, nay deserve that recognition as members of the great healing art so earnestly coveted.

In conclusion, believing that in a careful consideration of the subject matter of this paper, however imperfectly brought out, there will be recognized many of the conditions and circumstances favoring the "chemical theory of decay, but lacking the ability, as well as the limits of this paper—already too long—forbidding its discussion, I may be pardoned for presenting a few authoritative facts, or statements, which in connection with the foregoing, will be readily recognized by those who have given this subject any thought as bearing directly upon it.

The case cited by Prof. Spalding, in an article published in the *State Journal*, March, 1882, is just to the point; a girl, aged 12, teeth decaying rapidly, neither gold nor other fillings, notwithstanding thorough cleanliness, remaining any length of time, the saliva unexpectedly showing an *alkaline* reaction, other similar cases showing a like condition of the secretions of the mouth.

Dr. Watt, in the October, 1884, number of the *State Journal*, says: "It is possible for the lungs to give off ammonia in cases of uræmia, but find it in no *other* condition of the system."*

Marchand was the first to discover to a certainty, the presence of ammonia in the exhalations.

"In the pulmonary exhalations, small quantities of ammonia may always be recognized."—LEHMAN.

"Ammonia may always be found in the exhalations, and is sometimes increased by caries of the teeth, logwood paper being the best test of its presence, showing it when diluted 64 million times."—REULING.

"On the disintegration of nervous and muscular tissue, the albuminous constituents resolve themselves into two classes of compounds, one rich in carbon, and the other nitrogen, yielding by decomposition carbonic acid and ammonia respectively."—CARPENTER.

"Nitrogenous histogenetic substances may be classified into albuminous and gelatinous substances, which undergo putrefaction when exposed to the atmosphere, and in the presence of water, *carbonate*, and *sulphide* of *ammonia* always being among the products of this decomposition."—LEHMAN.

---

* It is Dr. Talbot who says this.—ED. REGISTER.

"Almost all histogenetic substances develop ammonia when treated with dilute acids or alkalies."—LEHMAN.

"Azotised organic matters, exposed to the air in contact with salifiable bases and water, yield salts and nitric acids, the base may even be ammonia."—ZUIELIN, from October No. of State *Journal.*

"In affections involving the nervous system, the saliva is never acid but often strongly alkaline."—LEHMAN.

"Mucous secretions are greatly increased in quantity by irritation of the mucous membrane and accompanied by an excess of albumen."—PAGET.

"Exudations from wounds and irritated surfaces do not differ in their constituents from those of the liquor sanguinis."—PAGET.

"Chloride of sodium introduced into the system, partially undergoes decomposition, whereby hydrochloric acid is produced."—CARPENTER.

"Hydrochloric acid does not, like lactic acid, decompose the phosphate of lime, but dissolves it out. This is one of the free acids frequently found in the saliva in its abnormal conditions and is also an ingredient of gastric juice."—WATT.

